# Editorial: Orthodox vs paradox: the roles of glycomics, genetics and beyond in immunity, immune disorders and glycomedicine

**DOI:** 10.3389/fimmu.2023.1305552

**Published:** 2023-10-16

**Authors:** Wei Wang

**Affiliations:** ^1^ The First Affiliated Hospital, Shantou University Medical College, Shantou, China; ^2^ School of Public Health, Shandong First Medical University & Shandong Academy of Medical Sciences, Shandong, China; ^3^ Beijing Key Laboratory of Clinical Epidemiology, School of Public Health, Capital Medical University, Beijing, China; ^4^ Centre for Precision Health, Edith Cowan University, Joondalup, Perth, WA, Australia

**Keywords:** paracentral dogma, sugar codes, glycomics, immunity, immune disorders, glycomedicine

The traditional ‘central dogma’ describes the flow of genetic information from DNA to RNA to protein. This process highlights the critical role of genes in living organisms. Nonetheless, ongoing immunological research coming to realize that emerging disciplines like glycomics and epigenetics are challenging the traditional viewpoint and extending the boundaries of the ‘central dogma’. This pivotal development has led to profound shifts in our understanding of how the immune system functions. Hence, one might wonder if there exists a ‘paracentral dogma’ that can offer answers to these revolutionary discoveries by taking sugars as the 3^rd^ life codes after nucleic acids and proteins, the 1^st^ and 2^nd^ of life codes for cellular materiality (1, 2).

The emergence of glycomics, particularly in the field of immunology, has revealed the biological functions of glycans and their key roles in the immune system (3). The richness and intricacy of glycans endow the immune system with extraordinary diversity and adaptability, impacting crucial processes within immune cells, encompassing signaling, interactions, and adhesion. Such innovative discoveries provide new perspectives for immunological research and bridge glycomics and immunology as well as genetics and epigenetics, contributing to a deeper insight into the functioning of the immune system (4).

Genetics and epigenetics play an integral role in immune-related disease research. Along with the co- and post-translation modifications, genetic variants significantly affect the functioning of the immune system, leading to the occurrence and progression of immune-related diseases. Investigating the relationship between genetic/epigenetics and immune diseases has become an important part of unraveling the mysteries of immunology. Intensive research in this area has provided us with key information on the diversity of the immune system and the omics basis of immune-related diseases (5).

This Research Topic, therefore, aims to integrate glycomics and genetics knowledge for a more comprehensive and in-depth understanding of the function and regulation of the immune system. This unique insight will contribute to the development of innovative disease therapy and vaccination. This Research Topic brings together important research findings from distinguished researchers and scientists globally. With this Research Topic, we hope to offer readers more opportunities to learn about the latest research and cutting-edge advances in this domain. We believe that we can open up new avenues for future research in glycomedicine and life sciences through such interdisciplinary collaboration and knowledge sharing.

In this Research Topic, we are pleased to present one Brief Research Report, four Review articles, and six Original Research articles. A diverse array of pivotal subtopics is explored:

- Synergistic regulation of Notch signaling by different O-glycans promotes hematopoiesis (Tanwar and Stanley).
- Review breakthrough of glycobiology in the 21^st^ century (Mahara et al.)
- Blood DNA methylation marks discriminate Chagas cardiomyopathy disease clinical forms (Brochet et al.)
- Effects of low-calorie and different weight-maintenance diets on IgG glycome composition (Deriš et al.)
- The IgG glycome of SARS-CoV-2 infected individuals reflects disease course and severity (Siekman et al.)
- Identification and validation of IgG N-glycosylation biomarkers of esophageal carcinoma (Pan et al.)
- Glucose metabolism and glycosylation links gut microbiota to autoimmune diseases (Wang et al.)
- Alterations of m6A RNA methylation regulators contribute to autophagy and immune infiltration in primary Sjögren’s syndrome (Cheng et al.)
- N-glycosylation and inflammation; the not-so-sweet relation (Radovani and Gudelj)
- Glycometabolism reprogramming of glial cells in central nervous system: Novel target for neuropathic pain (Kong et al.)
- Cytokines in the immune microenvironment change the glycosylation of IgG by regulating intracellular glycosyltransferases (Cao et al.)

These articles focus on the latest advancements in the field of glycomics, genetics, immunology and glycomedicine, addressing topics including:

- Glycobiology and the immune system: how glycobiology interacts with the immune system and how they work together to influence health and disease.
- DNA methylation and disease: how DNA methylation plays a role in different clinical cases, especially in the identification of Chagas cardiomyopathy disease.
- Diet, disease and IgG glycome composition: how diet affects the IgG glycome composition and its implications for disease.
- Inflammation, autoimmune disease, and glucose metabolism: the link between autoimmune disease, inflammation, and glucose metabolism, with particular reference to interactions with gut microbiota.
- RNA methylation, autophagy and immune infiltration: how RNA methylation affects autophagy and immune infiltration, especially in primary Sjögren’s syndrome.
- Cytokines and glycosylation in the immune microenvironment: how cytokines in the immune microenvironment affect IgG glycosylation by regulating glycosyltransferases.

Echoing the presuppositions of the ‘paracentral dogma-supporting the central dogma with sugar codes”, this Research Topic not only broadens the scope of central dogma, but also lays a solid foundation for future research in immunology and glycomedicine. We further contribute to the development of emerging disciplines by offering insights into the interrelationships between glycobiology, immunology and genetics. We expect that this Research Topic will stimulate more collaborations among these fields and lead to new breakthroughs and innovations for the scientific community. This series of articles will enable us to promote ‘glycomedicine’ and it’s role in maintaining health and addressing immune diseases (1, 6, 7). Relevant findings will shed valuable light on the realization of preventive, predictive, and precision medicine.

## Author contributions

WW: Conceptualization, Funding acquisition, Writing – original draft, Writing – review & editing.
